# Editorial: Deciphering the role of macrophages in lung homeostasis and diseases

**DOI:** 10.3389/fimmu.2022.1105304

**Published:** 2022-12-22

**Authors:** Zhilong Jiang

**Affiliations:** Department of Pulmonary Medicine, Zhongshan Hospital, Fudan University, Shanghai, China

**Keywords:** macrophage, lung fibrosis, asthma, acute lung injury, immune response

Macrophages are heterogeneous innate immune cells and play important roles in the development of various inflammatory lung diseases. The collected articles in this Research Topic cover the complicated function of lung macrophages in various lung diseases, including lung fibrosis, asthma, and acute lung injury (ALI). There are two review articles and one original article that cover the role of macrophages in lung fibrosis. Three original articles investigate the roles of different macrophage subtypes in asthma and ALI. These articles provide insight into the different immune functions of macrophages in lung fibrosis, asthma, and ALI.

The results reported by Bhattacharyya et al. show that Connexin 43 hemichannels affect ATP efflux in macrophages. The deletion of Connexin 43 hemichannels in macrophages reduced ATP efflux and subsequently reduced cytosolic calcium responses in co-cultured fibroblasts through the ATP receptor, P2rx4. Fibroblast-specific deletion of P2rx4 decreased lung fibrosis and collagen expression in lung fibroblasts of a mouse model with bleomycin-induced lung fibrosis, indicating the potent role of Connexin 43/P2rx4 interaction in driving polarization of pro-fibrotic subtype macrophages and subsequent development of lung fibrosis. The other two review articles related to lung fibrosis are respectively reported by Gu et al. and Wen et al. Gu et al. discuss the complicated and distinct roles of lung interstitial macrophages in the development of lung fibrosis. Autophagy plays an important role in clearing protein aggregates, damaged organelles, and invading pathogens. In the mini-review article, Wen et al. discuss the role of autophagy in macrophage polarization and lung fibrosis, demonstrating the protective role of appropriate macrophage autophagy in suppressing cell apoptosis, inflammation, and lung fibrosis through promoting the polarization of anti-inflammatory M2-like macrophages.

The role of lung macrophages in asthma has been widely investigated. Hernandez-Ramirez et al., in this article collection, report that a major allergen of mold Alternaria alternata, Alt a 1, promotes allergic asthma in mice through interaction with TLR4 in macrophages, accompanied by increased tissue remodeling and infiltration of eosinophils and macrophages. Another article by Wu et al. in this collection reveals the significant role of methyl-CpG-binding domain 2 (MBD2) in asthma. MBD2 expression was upregulated in macrophages of asthmatic mice and patients. Macrophage-specific knock-out and knock-down of MBD2 effectively attenuate ovalbumin-induced allergic airway inflammation, associated with the reduced polarization of M2 subtype macrophages, indicating the role of MBD2 in driving M2 cell-biased polarization and development of asthma.

Acute respiratory distress syndrome (ARDS) presents as uncontrolled lung inflammation in the whole lungs with massive exudes in the airspaces of the alveoli. Alveolar macrophages are first-line innate immune cells against invading pathogens and other insults, exhibiting both protective and inflammatory properties in the pathogenesis of ARDS. Signal regulatory protein-alpha (SIRP-alpha) is a transmembrane glycoprotein located on the surface of myeloid cells. SIRP-alpha suppresses macrophage phagocytosis after interaction with multimeric surfactant protein D (SP-D) (1). Shen et al., in this collection, discover that the membrane-bound SIRP-alpha can be released into the bronchoalveolar lavage (BAL) of mice with lipopolysaccharides (LPS)-induced ALI. A high level of soluble SIRP-alpha protein was observed in BAL of ALI mice, and that would be considered as a potential new biomarker in the diagnosis and evaluation of ARDS/ALI. A blockade of the soluble SIRP-alpha activity in ALI BAL reduced macrophage activation, indicating the pro-inflammatory property of soluble SIRP-alpha. Further study in SIRP-alpha knockout mice confirmed that the lack of SIRP-alpha significantly reduces ALI severity in mice, accompanied by reduced infiltration of neutrophils and expression of pro-inflammatory cytokines. The lack of SIRP-alpha in bone marrow-derived macrophages (BMDMs) improves macrophage phagocytosis, associated with suppressed Src homology 2 domain-containing protein tyrosine phosphatase 1 (SHP-1), but improves the activation of the signal transducer and activator of transcription-3 (STAT3) and STAT6. The study for the first time provided insight into the role of SIRP-alpha in the development of ALI.

Altogether, the articles in this Research Topic portray the multiple roles of macrophages in different animal models of lung diseases and highlight the need to better understand the molecular mechanisms of complicated macrophage function in different lung diseases through interaction with other types of cells under pathological conditions. The modulation of macrophage function by molecular intervention would be a promising therapeutic approach in the treatment of various inflammatory lung diseases.

## Author contributions

The author confirms being the sole contributor of this work and has approved it for publication.
